# Auswirkungen der Online-Lehre auf die Psychotherapieausbildung. Eine Mixed-Method-Studie an der Sigmund Freud PrivatUniversität Wien

**DOI:** 10.1007/s00729-023-00223-1

**Published:** 2023-06-14

**Authors:** Adalet Akgün, Roxane Forghani, Jutta Fiegl, Elitsa Tilkidzhieva

**Affiliations:** 1 Fakultät Psychotherapiewissenschaft, Institut für Ausbildungsforschung, Sigmund Freud PrivatUniversität Wien, Freudplatz 1, 1020 Wien, Österreich; 2 Fakultät Psychotherapiewissenschaft, Sigmund Freud PrivatUniversität Wien, Freudplatz 1, 1020 Wien, Österreich

**Keywords:** Psychotherapieausbildung, Psychotherapieforschung, Online-Lehre, Onlineausbildung, COVID-19, Psychotherapy training, Psychotherapy research, Online teaching, Online training, COVID-19

## Abstract

Die pandemiebedingte Umstellung auf die Online-Lehre im Jahr 2020 stellte die Psychotherapieausbildung vor neue Herausforderungen, vor allem, weil der persönliche Kontakt und die Praxiserfahrung für die Ausbildung in diesem Beruf so wesentlich sind. Ausgehend von den Erfahrungen der Auszubildenden wurden in dieser explorativen Querschnittsstudie (Mixed-Methods-Design) die Auswirkungen der Online-Lehre auf die Psychotherapieausbildung an der Sigmund Freud PrivatUniversität Wien untersucht. Die Ergebnisse zeigen, dass die Auszubildenden – trotz einiger Herausforderungen – im allgemeinen positive Erfahrungen mit der Online-Lehre gemacht haben und in der Lage waren, sich an die neuen Umstände anzupassen. Gleichzeitig zeigte sich, dass die psychotherapeutische Ausbildung Online nur bedingt möglich ist, da u. a. die in der Präsenz gemachten Erfahrungen nicht vollständig Online erlebt werden konnten.

## Abstract (en)

The 2020 pandemic-induced shift towards online teaching presented new challenges to psychotherapy training, primarily because interpesonal contact and practice experiences are so essential for training in this profession. In this explorative cross-sectional study (mixed-methods design) the impact of online teaching on the psychotherapy training at Sigmund Freud University Vienna was investigated based on the experiences of trainees. Results indicate that despite some challenges, trainees generally had positive experiences with online teaching and were able to adapt to the new circumstances. It became apparent that the online psychotherapeutic training is only possible to a limited extent, since, among other things, the experiences made in presence could not be felt entirely online.

## Einleitung

Der Ausbruch von COVID-19 brachte viele Herausforderungen mit sich, vor allem für die universitäre Ausbildung (Aucejo et al. 2020; Aguilera-Hermida 2020; Mishra et al. 2020). In der ersten Hälfte des Jahres 2020 waren Hochschulen auf der ganzen Welt gezwungen, vollständig auf Online-Lehre umzustellen. Lehrende, die mit der Online-Lehre bis dato nicht vertraut waren, haben ihre Lehrprogramme umgestaltet, um ihre Studierenden in einer absoluten Online-Umgebung zu unterstützen. Dies erforderte eine Umstellung der didaktischen Praxis sowie den Einsatz neuer Technologien.

Jüngste Meta-Analysen belegen zwar, dass der Studienerfolg zwischen Präsenz- und Online-Lehre vergleichbar, letzeres aber „mit mehr Lernzeit, Ressourcen und Interaktionen zwischen Lernenden konfundiert“ ist (Krammer et al. 2020, S. 340). Die Ersetzung von Präsenzveranstaltungen durch Online-Äquivalente führte dabei zu einigen Beeinträchtigungen in bestimmten Ausbildungsbereichen (Ferrel und Ryan 2020).

Negative Auswirkungen auf die Gesundheit sind zusätzliche Faktoren, die die Online-Lehre beeinträchtigen. So zeigen Brakemeier et al. (2020), dass seit Ausbruch der COVID-19 Pandemie eine globale Zunahme von psychischen Problemen, insbesondere von Ängsten, Depressionen und Stress zu verzeichnen sind. Irawan et al. weisen in diesem Zusammenhang mit Bezug auf die Online-Lehre darauf hin, dass beim Online-Lernen Stress hervorgerufen wurde. Gleichzeitig gaben Studierende an, aufgrund der Umstellung auf die Online-Lehre unter Stimmungsschwankungen, Reizbarkeit und Schlafstörungen zu leiden (Irawan et al. 2020).

Die Online-Lehre stellte in diesem Sinne eine besonders große Herausforderung dar: nicht nur im Hinblick auf die abrupte Umstellung des Lehrangebots bei der Sigmund Freud PrivatUniversität (SFU) Wien, sondern auch für die psychotherapeutische Ausbildung bzw. das Studium insgesamt, da neben dem theoretischen und praktischen Wissenserwerb zwischenmenschliche Begegnungen, intensive Selbsterfahrung und -reflexion, unmittelbares Feedback und persönlicher Kontakt essenziell und erforderlich sind (Laubreuter 2020).

Vor diesem Hintergrund galt das Forschungsinteresse dieser explorativen Studie der Umstellung und den Auswirkungen von Online-Lehre auf die psychotherapeutische Ausbildung an der Fakultät für Psychotherapiewissenschaft der SFU Wien. In der vorliegenden Studie, die nur ein Teil eines größeren Studienprojekts darstellt, soll der Forschungsfrage nachgegangen werden, *inwiefern der Erwerb einer psychotherapeutischen Ausbildung über die Online-Lehre möglich ist.* Zudem wurden folgende Fragestellungen formuliert: Wie haben die Auszubildenden die Umstellung auf die Online-Lehre erlebt? Und wie hat sich die Online-Lehre an der SFU Wien auf die psychotherapeutische Ausbildung ausgewirkt? Was waren förderliche und/oder hinderliche Aspekte?

## Studiendesign

Der Studien-Typ ist eine explorative Querschnittsstudie im Mixed-Methods-Design. Die Datengewinnung setzt sich aus einem quantitativen Fragebogen und anschließend durchgeführten qualitativen Interviews im Rahmen von Fokusgruppen zusammen.

Zur quantitativen Erhebung wurde ein Fragebogen erstellt, in dem soziodemografische Daten, Fragen in Bezug auf die Online-Lehre sowie unterschiedliche Aspekte der Psychotherapieausbildung an der SFU Wien berücksichtigt wurden. Aufgrund des explorativen Charakters der Studie wurden auch einige validierte Skalen verwendet, wie z. B. „Online Learning Readiness Scale“ (OLR-Skala) von Hung et al. (2010) (Dimensionen: Computer‑/Internet-Selbstwirksamkeit, selbstgesteuertes Lernen, Lernkontrolle, Lernmotivation und Selbstwirksamkeit der Online-Kommunikation) und Aspekte der Online-Lehre (Krammer et al. 2020) (Didaktik, Kommunikation, organisatorische Faktoren der Umstellung). Die Teilnahme an der Studie war freiwillig und anonymisiert.

Ergänzend zum quantitativen Fragebogen wurden Fokusgruppen gebildet, um tiefgehende Einblicke hinsichtlich der Auswirkungen der Online-Lehre auf das Erleben der Auszubildenden zu erhalten. Hierzu wurden Semestergruppen (Propädeutikum und Fachspezifikum) gebildet.^1^ Mithilfe eines halbstrukturierten Interviewleitfadens wurde u. a. die Umstellung auf die Online-Lehre, Erfahrungen im praktischen Bereich und das Erlernen des psychotherapeutischen Berufs im Online-Setting erfragt. Die Gespräche wurden nach Einwilligung der Teilnehmenden aufgezeichnet, anonymisiert und transkribiert.

### Stichprobe

Insgesamt nahmen 105 Auszubildende an der quantitativen Umfrage teil. Davon waren 75 % (*N* = 79) Frauen und 25 % (*N* = 26) Männer. Das Durchschnittsalter betrug 29,71 Jahre (SD = 10,77, Min = 18, Max = 71). Zum Zeitpunkt der Erhebung befanden sich 46 % (*N* = 48) im Propädeutikum und 54 % (*N* = 57) im Fachspezifikum (u. a. Individualpsychologie 11 % (*N* = 12), Integrative Gestalttherapie 10 % (*N* = 11), Verhaltenstherapie 9 % (*N* = 9), Psychoanalyse 8 % (*N* = 8)). Überdies gaben 56 % (*N* = 59) Personen an, berufstätig zu sein.

An den vier qualitativen Fokusgruppen nahmen insgesamt zwölf Auszubildende teil, davon befanden sich jeweils sechs im Propädeutikum und Fachspezifikum. Unter den fachspezifischen Methoden waren u. a. die Individualpsychologie, die Systemische Familientherapie, die Integrative Gestalttherapie und die Psychoanalyse vertreten.

### Auswertung

Die erhobenen quantitativen Daten wurden mithilfe von SPSS 27.0 deskriptiv-statistisch ausgewertet, dafür wurden die ermittelten Werte jedes einzelnen Items berechnet.

Die qualitativen Daten wurden mithilfe von MAXQDA 2020 analysiert und mit der qualitativen Inhaltsanalyse nach Mayring (2003) ausgewertet. Mit der deduktiven Vorgehensweise wurden aus dem Transkriptmaterial induktive und deduktive Kategorien mit jeweiligen Kodierregeln und Ankerbeispielen erstellt.

## Ergebnisse

### Wie haben die Auszubildenden die Umstellung auf die Online-Lehre erlebt?

Die Mehrheit der Auszubildenden (*N* = 74, 70 %) hatten vor der Umstellung keine Erfahrungen mit der Online-Lehre gehabt. Weiteres gaben 65 % (*N* = 68) an, dass sie sich darauf gut vorbereiten konnten, wobei 57 % (*N* = 60) der Auszubildenden die technischen Anleitungen der Universität zur Vorbereitung als wenig geeignet empfanden.

Um die Umstellung auf die Online-Lehre und die Erfahrungen der Auszubildenden zu untersuchen, wurde zusätzlich die OLRS-Skala (Hung et al. 2010) angewendet. Die Analyse zeigt, dass die durchschnittlichen Werte für alle fünf Dimensionen über dem Mittelwert von 3,5 liegen. Das impliziert, dass die Auszubildenden eine große Bereitschaft in den Dimensionen Computer- und Internet-Selbstwirksamkeit (M = 4,76; SD = 1,26), Selbstgesteuertes Lernen (M = 4,28; SD = 1,41) und Lernmotivation (M = 3,87; SD = 1,55) aufzeigen. Die niedrigsten Werte liegen auf den Skalen Lernkontrolle (M = 3,83; SD = 1,61) und Selbstwirksamkeit der Online-Kommunikation (M = 3,65; SD = 1,53), was darauf hindeutet, dass die Aufmerksamkeit eingeschränkter ist und Hemmungen bei virtuellen Diskussionen entstehen.

In den Fokusgruppen wurde deutlich, dass vor allem der Pandemiebeginn Auszubildende verunsicherte und die Umstellungsphase ein gewisses Unbehagen auslöste. Allerdings waren die regelmäßigen Aufklärungs- und Informationsmails seitens der SFU Wien (über den Verlauf etc.) hilfreich, sodass Auszubildende sich „nicht alleingelassen“ gefühlt haben.

Die größte Herausforderung war der mühselige Umstieg, der mit Problemen bei der Semesterplanung und Informationsbeschaffung einherging, da u. a. Lehrveranstaltungen kurzfristig geändert wurden. Weiters wurde berichtet, dass die Nutzung unterschiedlicher Plattformen eine große Belastung für die Auszubildenden darstellte, da diverse Plattformen für verschiedenste Formen von Mitteilungen benutzt wurden (A5, Cloud, Moodle, Outlook, MS Teams etc.). Obwohl der größte Teil der Auszubildenden gut bis sehr gut mit der Nutzung von digitalen Plattformen zurechtkam, mussten sie die benötigten Informationen selbst „zusammensuchen“. Zu den häufig verwendetsten Plattformen zählen u. a. MS-Teams und Zoom, womit die Auszubildenden gute bis sehr gute Erfahrungen gemacht haben.

Wenngleich die Umstellung nicht reibungslos war und mit gewissen Belastungen einherging, so haben die Auszubildenden insgesamt ihre Zufriedenheit zum Ausdruck gebracht, da sie froh und dankbar dafür waren, die Ausbildung trotz der Pandemie fortsetzen zu können.

### Wie hat sich die Online-Lehre auf die psychotherapeutische Ausbildung an der SFU Wien ausgewirkt?

**Figure 1:**
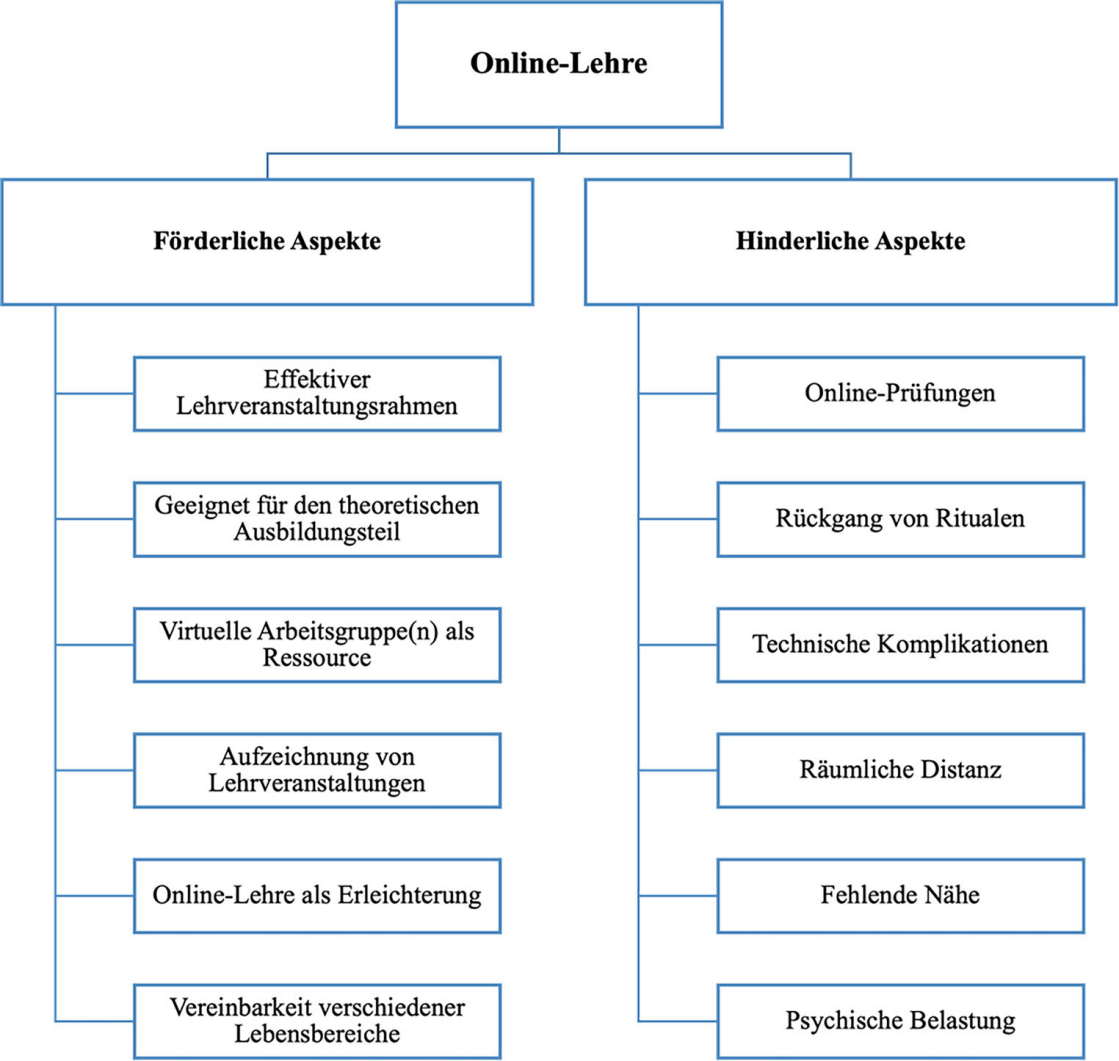

Die Auswertung hat gezeigt, dass die pandemiebedingten Änderungen die nachfolgenden Aspekte die Ausbildung beeinträchtigt haben. Qualitativ wurden u. a. zwei Hauptkategorien gebildet: Online-Lehre und Lehrende:r als Wirkfaktor, unterteilt in jeweils förderliche/hinderliche und positive/negative Aspekte (siehe Abb. 1).

#### Förderliche Aspekte der Online-Lehre

Die Online-Lehre wurde insgesamt positiv wahrgenommen. Sie wurde von den Auszubildenden als „revolutionäre Lehrform“ bezeichnet, die viele positive Aspekte mit sich bringt:

- *Effektiver Lehrveranstaltungsrahmen:* Für die Mehrheit der Befragten ist die Online-Lehre willkommen. Online-Lehrveranstaltungen waren dann beliebt, wenn regelmäßige Pausen (5–10 min) eingelegt worden sind oder sich die Pausen nach dem Wunsch der Auszubildenden gerichtet haben und nicht nach den obligatorischen zwei Unterrichtseinheiten am Stück. Dadurch konnte eine hohe Aufmerksamkeit und Motivation aufgebracht werden.
- *Geeignet für den theoretischen Ausbildungsteil:* Theoretisch wurde der Inhalt „fantastisch vermittelt“. Hierein spielt die Nutzung diverser Medien eine große Rolle, da diese als besonders hilfreich und notwendig für die Aufmerksamkeit und Konzentration erachtet wurde. Beispielsweise wurden anschaulich vorgestellte Falldarstellungen (z. B. durch Filmsequenzen etc.) und dazugehörige Rollenspiele als sehr hilfreich und nachvollziehbar für die eigene Praxis empfunden.
- *Virtuelle Arbeitsgruppe(n) als Ressource: *Kontakt zu Kommiliton:innen und Arbeitsgruppen wurden als förderlich beschrieben. Der soziale Kontakt gestaltet sich nun anders, allerdings lief es besser als angenommen und ein gutes Gruppengefühl konnte mit zunehmender Gewöhnung an die digitale Infrastruktur auch Online geschaffen werden – v. a. via WhatsApp-Semestergruppen. Diese Social-Media-Plattformen haben soziale Interaktionen begünstigt, sodass ein guter kommunikativer Austausch zwischen den Arbeitsgruppen entstand. Schließlich konnten die Auszubildenden auch virtuell ein besseres Feingefühl und Gespür für die Anwesenden entwickeln.
- *Aufzeichnung von Lehrveranstaltungen:* Aufgezeichnete Lehrveranstaltungen wurden als „effektiver und intensiverer Wissenserwerb“ beschrieben, da sich Auszubildende durch diese Möglichkeit mit den Inhalten wiederholt und flexibler befassen konnten, vor allem wenn sie Konzentrationsschwierigkeiten während der Lehrveranstaltung hatten. Die Rezeption wesentlicher Passagen erhöht die Reflektion und das Verstehen des Materials, was „durch eine analoge Lehrveranstaltung nicht möglich“ sei.
- *Online-Lehre als Erleichterung: *Viele der Auszubildenden nahmen die Online-Lehre als Erleichterung wahr, da diese Lehrmodalität u. a. eine Zeitersparnis darstellt (69 %, *N* = 73). Vor allem berufsbegleitende Auszubildende hatten „viel mehr Raum und Zeit“ für sich, da sie die positiven Seiten des Online-Settings gut nutzen konnten (u. a. die räumliche Freiheit, sich von überall einloggen zu können), weshalb eine größere Frustrationstoleranz gegenüber der Online-Lehre aufgebaut werden konnte. Die Verschiebung des formalen Rahmens schaffte zudem die Möglichkeit, persönliche Interessen intensiver als zuvor zu verfolgen.
- *Vereinbarkeit von Privatleben, Beruf und Familie: *Die Online-Lehre ermöglichte für viele eine flexiblere Tagesplanung und damit eine guten Vereinbarkeit von Studium, Ausbildung, Familie, Beruf und anderen Tätigkeiten. Ein Großteil der Auszubildenden (76 %, *N* = 79) gab zu verstehen, dass die Online-Lehre einen einfacheren Zugang zur Teilnahme ermögliche (z. B. für alleinerziehende Elternteile).

Schließlich war die Online-Lehre für Auszubildende mit einem „total bewegten Alltag und Leben eher eine Erleichterung“. Die Auszubildenden können sich deshalb eine Hybrid-Lehre mit der dafür erforderlichen Vielfalt an didaktischen Methoden längerfristig gut vorstellen, weil u. a. Konzentrationsschwierigkeiten mit zunehmender Gewöhnung an die Online-Lehre minimiert wurden.

#### Hinderliche Aspekte der Online-Lehre

Mit der Umstellung auf die Online-Lehre kamen neben förderlichen auch hinderliche Aspekte hinzu.

- *Online-Prüfungen: *Höchst problematisch waren Online-Prüfungen während der ersten Phase nach der Umstellung, da es vorab keinen Probelauf gab. Die Auszubildenden konnten sich schwer darauf einstellen und waren (technikbedingtem) Stress ausgesetzt. Im Laufe des Semesters haben sie sich zwar an die Online-Prüfungsform anpassen können, allerdings wurde der Prüfungsstoff inhaltlich so weit ausgedehnt, sodass laut einigen Auszubildenden die Prüfung „nicht mehr in dem Zeitrahmen zu schaffen war“ und einen „Tipp-Wettbewerb“ darstellte.
- *Rückgang von Ritualen: *Die Befragten gaben an, dass sie bei der Umstellung unmittelbare Schwierigkeiten mit dem Setting der Online-Lehre hatten, da der „formale Rahmen verloren“ gegangen sei. Dadurch ließen sich Auszubildende schneller ablenken und beschäftigten sich eher mit alltäglichen Aktivitäten (Kochen, Essen, Putzen etc.), vor allem wenn die eigene Kamera während der Lehrveranstaltung nicht eingeschaltet war. Einhergegangen sind damit Konzentrationsschwierigkeiten und Müdigkeit. Da die Teilnahme an Lehrveranstaltungen in den informale Rahmen übergegangen ist (Sitzen im Pyjama, im Wohn‑/Schlafzimmer oder in der Präsenz von Familienmitgliedern) und die täglichen Routinen weggefallen sind (aufstehen, duschen, anziehen, wegfahren, im Gebäude ankommen, im Saal sitzen), fehlte die Motivation zum Studium, das Zugehörigkeitsgefühl sowie (teilweise) der Sinn des Studiums.
- *Technische Komplikationen: *Internetstörungen oder technische Probleme stellten auch eine Herausforderung dar, weshalb eine „gewisse Frustrationstoleranz“ benötigt wurde, um die erforderliche Aufmerksamkeit im Online-Setting aufzubringen.
- *Räumliche Distanz: *Der anwesende Körper während der Präsenz-Lehre stellt eine ganz andere Wirkung dar. Da das Beobachten der Körpersprache und das Hören von Zwischentönen durch das Online-Format erschwert wurde, war es mühseliger, die Freude des anderen als Ganzes zu erleben. „Zwischenmenschliches [kann] schlecht digital transportiert“ werden, weshalb das Wahrnehmen, Hineinfühlen und das Verstehen des anderen eine gewisse „Imagination/Vorstellungskraft“ benötigt.
- *Fehlende Nähe: *Da die informellen Kaffeepausen und Unterhaltungen ausfielen, fehlte der direkte Austausch unter Kolleg:innen. Die Mehrheit (80 %, *N* = 76) empfindet im Präsenzmodus ein höheres Gemeinschafts- und Zugehörigkeitsgefühl. Auszubildende, deren Ausbildungsbeginn mit der pandemiebedingten Schließung zusammenfiel, standen vor dem Problem, einen direkten Anschluss an andere Kolleg:innen zu finden, weshalb zu Beginn teilweise kein gutes Gruppengefühl entstehen konnte. Zudem berichtete die überwiegende Mehrheit der Befragten (84 %, *N* = 88), dass ihnen der Austausch mit Lehrenden (64 %, *N* = 68) und Kolleg:innen (84 %, *N* = 88) gefehlt hat. Es wurde als hilfreich empfunden, wenn ein größerer digitaler Platz für den Austausch angeboten wurde (z. B. Öffnen des virtuellen Raums 15 min vor und nach der Lehrveranstaltung).
- *Psychische Belastungen: *Im ersten Jahr der Online-Lehre gaben 51 % (*N* = 54) der Befragten an, oft bis sehr oft psychischem Stress ausgesetzt gewesen zu sein, wobei einige zusätzlich oft bis sehr oft (u. a.) folgende Symptome aufwiesen: 30 % (*N* = 32) Stimmungsschwankungen; 27 % (*N* = 28) depressive Zustände; 26 % (*N* = 27) Schlafstörungen; 24 % (*N* = 25) Beziehungsschwierigkeiten; 19 % (*N* = 20) nicht-substanzgebundene Abhängigkeiten (z. B.: Internet‑, Spiel- oder Kaufsucht) und 18 % (*N* = 18) Angststörungen. Die Angst, dass sich durch die Umstellung die Ausbildung verlängern könnte, erzeugte auch einen psychischen und finanziellen Druck, der zusätzlich durch die fehlende soziale Interaktion größer wurde. Des Weiteren gab ein:e Interviewte:r an, einen kleinen Wohnraum gehabt zu haben, sodass der strenge Lockdown im Winter ihn/sie psychisch schwer belastete.

Der Großteil der hinderlichen Aspekte bezieht sich vor allem auf den Beginn der Umstellung. Mit der Gewöhnung an das neue Format gingen positive Effekte einher, wie etwa eine Erleichterung für den beruflichen Alltag und Entwicklung von Bewältigungsstrategien. Hierzu wurde als Strategie etwa eine aktive Auszeit vom Computer durch Sport, Gang in die Natur und das Nachgehen von Hobbies herangezogen. Zudem wurde das Einhalten von täglichen Routinen, eine ausgewogene Ernährung und der Einsatz von Entspannungsmethoden als sehr hilfreich empfunden.

### Lehrende:r als Wirkfaktor

**Figure 2:**
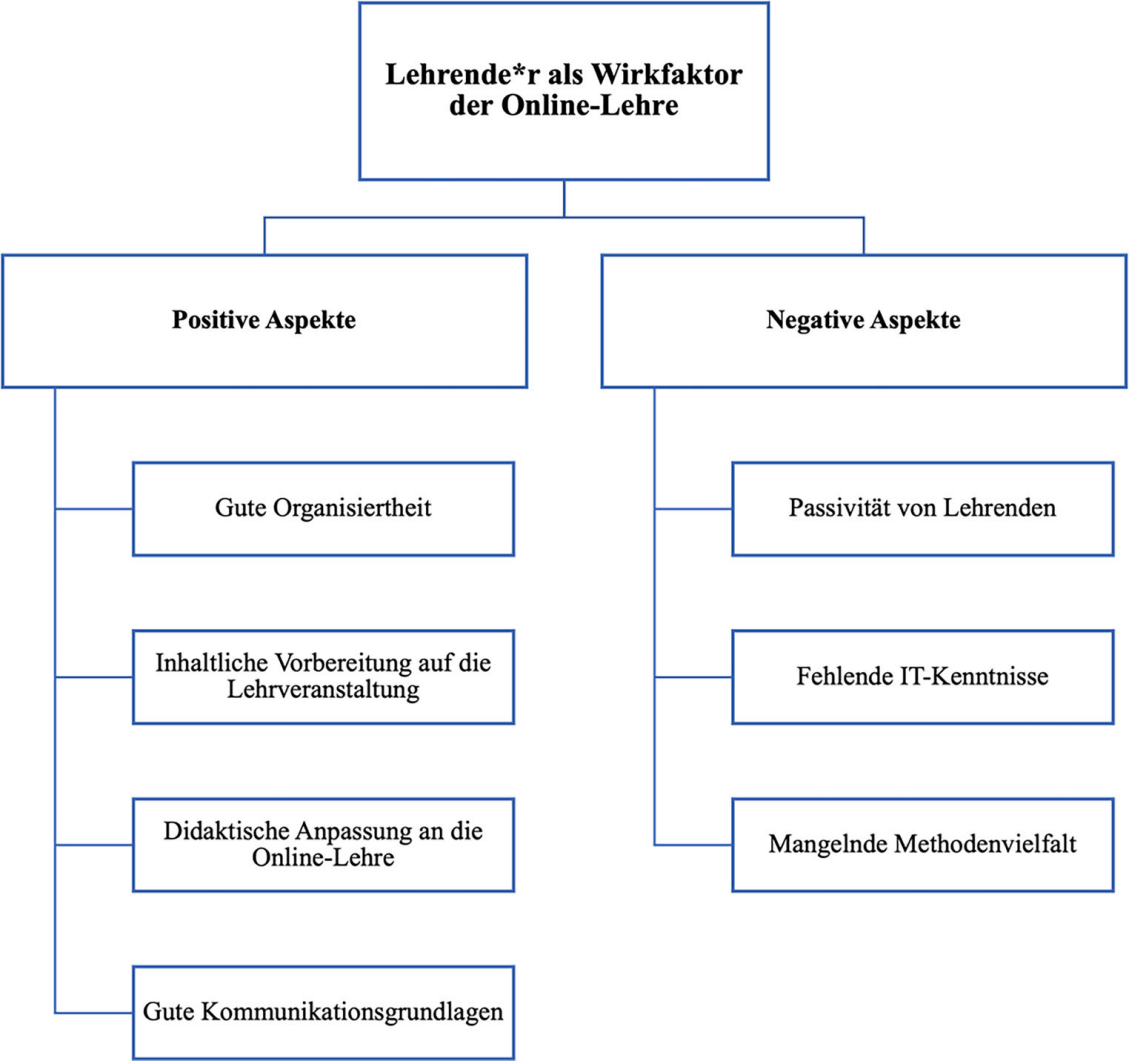

Aus dem qualitativen Datenmaterial wurde die deduktive Kategorie *Lehrende:r als Wirkfaktor* gewonnen, da sie als Akteur:innen die Online-Lehre unmittelbar beeinflussen. Die Umstellung stellte Lehrende vor neue Herausforderungen, wobei diese in positive und negative Aspekte unterteilt werden können (siehe Abb. 2).

#### Positive Aspekte

Auszubildende waren vor allem zufrieden mit der Online-Lehre, wenn Lehrende …:

- *gut organisiert waren: *Hierzu wurde genannt, dass sich viele „Lehrende mächtig Mühe gegeben“ haben. Sie haben u. a. die organisatorischen Problematiken durch ihre Flexibilität ausgleichen können.
- *inhaltlich gut vorbereitet waren: *Die Qualität nahm auch dadurch zu, wenn die Lehrinhalte nicht „runtergeleiert oder abgelesen“, sondern frei vorgetragen wurden. Dies steigerte die Aufmerksamkeit und Konzentration. Auch neue Formate wurden willkommen geheißen, selbst wenn die Technik nicht beim ersten Mal sofort funktioniert hat.
- *Online-Format didaktisch anpassten: *Auszubildende konnten sich bis zum Ende einer Online-Lehrveranstaltung gut konzentrieren, wenn viele Fallbeispiele oder Medien, wie Bilder, PowerPoints, Videosequenzen und Filmanalysen integriert wurden.
- *gute Kommunikationsgrundlagen schafften: *Es wurde sehr geschätzt, wenn Lehrende sich bemühten und gleichzeitig einen Raum der Offenheit schafften, dies habe sogar „organisatorische Mängel“ wettgemacht. So entstanden „sehr schöne Begegnungen“, die gleichzeitig auch „kleine Privatsphären“ schafften.

#### Negative Aspekte

Bei den negativen Aspekten bezogen sich die Auszubildenden vor allem auf die schlechte Konzeptualisierung bzw. mangelnde Anpassung an die Online-Lehre:

- *Passivität von Lehrenden: *Einige Befragte fanden es schwierig, wenn es den Auszubildenden während einer Online-Lehrveranstaltung frei stand, sich mit der Kamera dazu zu schalten. So entstand bei den Befragten der Eindruck, dass die aktive Teilnahme an der Lehrveranstaltung nicht obligatorisch ist, weil sie von den Lehrenden nicht dazu animiert wurden, sich aktiv einzubringen. Diese Form von Passivität von Lehrenden machte es umso schwieriger, sich in die Online-Lehrveranstaltung eingebunden zu fühlen.
- *Fehlende IT-Kenntnisse: *Das Online-Setting stellte auch eine Herausforderung für die Lehrenden dar, denn 60 % (*N* = 65) der Auszubildenden gaben an, dass den Lehrpersonen grundlegende IT-Kenntnisse fehlten, weshalb hierdurch eine höhere Belastung für Auszubildende aufkam.
- *Mangelnde Methodenvielfalt: *Durch die digitale Lehrveranstaltungsform hatten Auszubildende Schwierigkeiten darin, sich lange zu konzentrieren und gaben an, schnell müde bzw. gelangweilt zu sein (57 %, *N* = 60). Die Aufmerksamkeit ließ noch schneller nach, wenn Lehrende nicht nur Schwierigkeiten bei der Umsetzung der Online-Lehre hatten, sondern auch, wenn passende didaktische Methoden fehlten. 43 % (*N* = 45) der Befragten vertraten die Meinung, dass in den Online-Lehrveranstaltungen nicht ausreichend abwechslungsreiche Lernmethoden verwendet wurden. So hatten einige Auszubildende den Eindruck, dass sie in einigen Online-Lehrveranstaltungen mehr beigetragen hätten als die Lehrenden, da mehrere Lehrveranstaltungen so konzipiert wurden, dass Auszubildende Referate vortragen sollten und der Fokus bzw. der Inhalt der Online-Lehrveranstaltungen rein darauf begrenzt wurde. Dies wurde weder als förderlich noch als sinnvoll erachtet. Hier wurde u. a. der Wunsch geäußert, die Online-Lehrveranstaltungen aufgeschlossener, zugänglicher und differenzierter zu gestalten.

Auch wenn das Online-Format eine neue Herausforderung für Lehrende darstellte, so war nicht nur das Gelingen, sondern auch die Mitwirkung und der Wissenserwerb der Auszubildenden in Online-Lehrveranstaltungen maßgeblich von Lehrenden abhängig (Arghode et al. 2017).

## Konklusion

Die pandemiebedingte Umstellung auf die Online-Lehre 2020 stellte nicht nur Hochschulinstitutionen, sondern ebenso Lehrende vor neue, pädagogische Herausforderungen. In der vorliegenden Studie über die Auwirkungen von Online-Lehre auf die Psychotherapieausbildung bekräftigten sich die bereits bekannten hinderlichen Aspekte bezüglich der Online-Lehre, wie etwa ein höherer Aufwand zur Aufrechterhaltung der Konzentration und verhäufte psychische Belastungen (Irawan et al. 2020). Nichtsdestotrotz ließen sich ebensoviele förderliche Aspekte identifizieren, wie z. B. die Anwendung von innovativen, didaktischen Ansätzen und das Gelingen neuer Beziehungsformen (Krammer et al. 2020).

Insgesamt erwies sich die Online-Lehre für die Ausbildung zum/zur Psychotherapeut:in als gute Alternative und Erleichterung in der pandemiebedingten Situation. Sie bietet eine Flexibilität, die das Privatleben und den Beruf in Einklang bringen lässt, weshalb Auszubildende die Hybrid-Lehre teilweise als wünschenswert erachten.

Es zeigte sich, dass der Erwerb einer psychotherapeutischen Ausbildung über die Online-Lehre nur bedingt möglich ist, wie z. B. bei kurzen theoretischen Lehrveranstaltungen, Supervision oder Praktikumsreflexionen. Allerdings wurden Ausbildungsbereiche, die mit den zwischenmenschlichen Begegnungen verknüpft sind (Selbsterfahrung, Austausch, Übungen, direktes Feedback), überwiegend in Mitleidenschaft gezogen, da bei der virtuellen Übertragung „etwas gefehlt“ hat.

Das angepasste Ausbildungsangebot (Didaktik, erweitertes Sozialangebot, individuelle Unterstützung) an die pandemiebedingte Situation wurde von den Studierenden gut angenommen. Dennoch deutet die vorliegende Studie darauf hin, dass für die Psychotherapieausbildung der Präsenzmodus unerlässlich ist, da das Gespür für den Anderen und die Erfahrungen, die im Präsenzmodus gemacht wurden, Online nicht zur Gänze erlebbar waren.

## Footnotes

1 Die Absolvierung der psychotherapeutischen Ausbildung sieht in Österreich gesetzlich zunächst ein Propädeutikum (vier Semester) und ein darauffolgendes Fachspezifikum (mind. sechs Semester; je nach Methodenwahl) vor. Beide Ausbildungsabschnitte haben sowohl theoretische als auch praktische Ausbildungsanteile, wobei das Fachspezifikum die methodenspezifische Ausbildung der Psychotherapie ist.
